# Determinants of Glycosaminoglycan (GAG) Structure

**DOI:** 10.3390/biom5032003

**Published:** 2015-08-21

**Authors:** Kristian Prydz

**Affiliations:** Department of Biosciences, University of Oslo, Box 1066, Blindern OSLO 0316, Norway; E-Mail: kristian.prydz@ibv.uio.no; Tel.: +47-2285-6753

**Keywords:** Proteoglycans, glycosaminoglycans, PAPS, 3′-phosphoadenosine-5′-phosphosulfate, Golgi apparatus, secretory pathway, epithelial cells, linker region

## Abstract

Proteoglycans (PGs) are glycosylated proteins of biological importance at cell surfaces, in the extracellular matrix, and in the circulation. PGs are produced and modified by glycosaminoglycan (GAG) chains in the secretory pathway of animal cells. The most common GAG attachment site is a serine residue followed by a glycine (-ser-gly-), from which a linker tetrasaccharide extends and may continue as a heparan sulfate, a heparin, a chondroitin sulfate, or a dermatan sulfate GAG chain. Which type of GAG chain becomes attached to the linker tetrasaccharide is influenced by the structure of the protein core, modifications occurring to the linker tetrasaccharide itself, and the biochemical environment of the Golgi apparatus, where GAG polymerization and modification by sulfation and epimerization take place. The same cell type may produce different GAG chains that vary, depending on the extent of epimerization and sulfation. However, it is not known to what extent these differences are caused by compartmental segregation of protein cores *en route* through the secretory pathway or by differential recruitment of modifying enzymes during synthesis of different PGs. The topic of this review is how different aspects of protein structure, cellular biochemistry, and compartmentalization may influence GAG synthesis.

## 1. Proteoglycans

Proteoglycans (PGs) consist of a protein core that, during transport through the secretory pathway, acquires one or more usually negatively charged glycosaminoglycan (GAG) chains. The negative charge is conferred by acidic sugar residues and/or sulfate groups in various positions along the GAG chains [1]. The acidic sugars are hexuronic acids that alternate with amino sugars in repeated disaccharide units: glucuronic acid (GlcA), that may become iduronic acid (IdoA) upon C5 epimerization, which occurs in certain GAG domains during heparan sulfate (HS) and heparin synthesis, and in the conversion of chondroitin sulfate (CS) into dermatan sulfate (DS). The GAG chains possess sub-domains that allow biologically important interactions with a wide variety of regulatory proteins [2,3,4,5].

GAGs that extend from PG protein cores to which they are covalently attached have the ability to attract cations and to bind water molecules. Hydrated GAG gels have long been known to play an important role for the absorption of pressure changes in joints and tissues. In addition, certain patterns of epimerization and sulfation along GAG chains promote ionic interactions with growth factors and other signaling molecules, thus, regulating growth development and differentiation and also influencing immunological mechanisms. The discovery of such mechanisms has also increased the interest in PGs in what concerns cancer development, metastasis, and therapy regimes [6,7].

PG protein cores are not just scaffolds for GAG extension. More than 40 different protein cores have been identified, many of which have been grouped according to their protein domains [8]. Such domains may be engaged in a number of different interactions and many of the protein cores display variants resulting from alternative splicing. Several reports show that PG protein cores may influence the type and modification patterns of the subsequently attached GAG chains, but how the information is transmitted from the protein core to the enzymes engaged in polymerization and modification of GAGs is not clear. One suggested mechanism is through sulfation and phosphorylation of sugar units of a linker tetrasaccharide that attaches GAG chains to a modification site of a protein core. The picture is complicated by the fact that the same protein core can acquire different types of GAG chains in different cell types, showing that GAG modification for a particular protein core may be cell type and tissue specific. For instance, the PG Serglycin is modified by CS chains, often sparsely sulfated in most cell types where it is expressed, but obtains highly sulfated heparin chains in mast cells [9].

**Figure 1:**
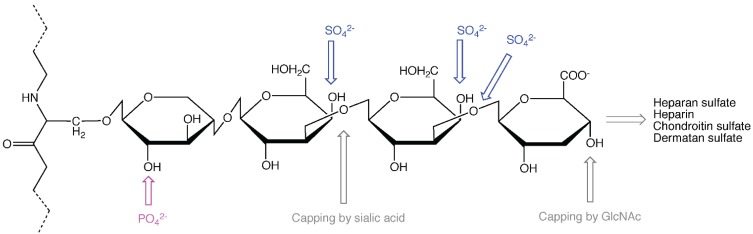
A heparan sulfate (HS)/heparin or chondroitin sulfate (CS)/dermatan sulfate (DS) attachment site in a proteoglycan (PG) protein core with a linker tetrasaccharide. Sulfation, phosphorylation and capping sites are indicated.

Apart from keratan sulfate GAGs that originate from typical glycoprotein modification sites [10], CS, DS, HS and heparin GAGs all extend from the serine residue of ser-gly sites in the protein core through the common linker tetrasaccharide (Figure 1), consisting of xylose [11], galactose, galactose, and glucuronic acid (GlcA). Addition of a fifth sugar, which is an acetylated amino sugar, will decide whether the GAG chain becomes HS/heparin or CS/DS. In the former cases (HS/heparin), the amino sugar is *N*-acetyl-glucosamine (GlcNAc), while in the latter cases (CS/DS) it is *N*-acetyl-galactosamine (GalNAc). In all these cases the amino sugars alternate with GlcA in an enzyme-catalyzed polymerization process resulting in long, linear GAG chains, consisting of disaccharide units that may undergo extensive modification. Heparin is more highly sulfated than HS and contains in addition some GlcN/NS carrying 3-*O*-sulfate, which is usually absent in HS. DS is generated from CS, firstly by epimerization of GlcA into IdoA, followed by sulfation in distinct positions [12].

An underlying question is how and why some GAG modification sites acquire HS chains, while others give rise to CS chains; and why some of these GAGs undergo particular modification regimes, for instance by conversion into DS, while CS GAGs attached to other protein cores are not subjected to the same changes?

In defined cellular systems, certain protein domains have been shown to promote HS synthesis, while deletions or site-directed mutagenesis has resulted in a decrease in the HS content, and instead an increased amount of CS modification. To my knowledge, there are no examples of protein domains that promote CS synthesis, which can be deleted or mutagenized to give more HS modification of a given PG protein core. This is one of the premises for the suggestion that CS modification might occur by default at sites that are not utilized for HS synthesis, as shown for embryonic stem cells that were made unable to synthesize HSPG, where CS synthesis compensates for some of the functions [13]. However, it may be shown possible to produce protein-free heparan sulfate chains *in vitro*, extending from the xyloside template GlcA-Gal-*O*-C_2_H_4_NH-benzyloxycarbonyl or from the protein α-thrombomodulin (which normally acquires CS chains), in the presence of the HS polymerizing enzymes EXT1 and EXT2 and the substrates UDP-GlcNAc and UDP-GlcA [14], indicating that HS synthases do not have a strict protein core requirement.

## 2. The Role of the Protein Core

PGs of the Glypican family are primarily modified by HS chains [15]. Glypican-1 expressed in CHO and COS cells requires the globular extracellular domain to become predominantly (90%) modified by HS chains. Removal of this domain, results in 90% CS modification, while the relative amount of HS chains may also be reduced by amino acid changes, up to 70 amino acids away from the GAG attachment sites, indicating that protein domains far away from the GAG sites play a role. Transfer of the globular domain of Glypican-1 significantly increased the relative amount of HS chains in both of the PGs Betaglycan and Decorin, supporting the view that this domain promotes HS modification [16]. Expression of chimeras of protein A with Betaglycan or Syndecan-1 showed that a nearby cluster of acidic amino acids and an adjacent tryptophan residue stimulate HS synthesis [17,18]. Repetitive ser-gly sequences also favor HS modification [19]. Similar observations were made for Perlecan [20], however, acidic amino acids are also found nearby typical CS sites in a number of PGs [21].

The domains of the protein core have been shown to have a greater influence on the detailed structure of CS chains than on that of HS chains in murine mammary gland epithelial cells. While the HS chains of Syndecan-1 and Syndecan-4 could not be distinguished, CS chains attached to these two PGs were structurally and functionally distinct [22], indicating that the CS chains are protein core specific and the HS structure could be more cell type specific. Several cell types have been reported to synthesize both lectican PGs, which are mainly modified by CS chains, and small leucine-rich PGs that mostly carry DS chains. The extent of C5 epimerization was shown to depend on motifs in the protein cores of Decorin (high incidence) and CSF-1 (low incidence), when full length DNA constructs and chimera coding these PGs were expressed in 293 HEK cells [23]. A more recent study showed that fusion PGs with high and low incidence of epimerization did not co-localize and that CSF-1 contained a protein domain (TNWVP) that prevented C5 epimerization and, thus, DS synthesis. A trp (W) to leu (L) change in this domain increased the IdoA content to 12%–16% [24]. CS and DS GAG chains may exist as separate entities, but can also form hybrid structures along the same GAG chain [25,26]. Over the years, more detailed information about the distribution of CS and DS domains has been obtained, and also on their tissue and regional differences, for instance in the brain [12,27]. Still, how domains of different modification status are made along the GAG chains, quite distantly from the protein core, is among the unanswered challenges of PG biology.

## 3. Modification of the Linker Region

Addition of a tetrasaccharide linker (xylose-galactose-galactose-GlcA) onto a serine residue in the protein core is the obligatory start of all CS/DS and HS/heparin GAG chains [28,29,30]. While GAG polymerization takes place in the lumen of the Golgi apparatus, synthesis of the linker region has been proposed to start in a pre-Golgi compartment, either at endoplasmic reticulum (ER) exit sites [31,32,33] or in the ER-Golgi intermediate compartment [34], while yet other researchers have localized the xylosyltransferases (I and II) to the *cis*-Golgi region [35,36]. What decides whether a particular linker region gives rise to a CS/DS chain or an HS/heparin chain has been subject to both speculations and experiments. Sometimes structures in the underlying protein core have been shown to play a role, but how the information is transmitted to the synthesis machinery is unclear. Modification of the linker xylose by phosphorylation at C-2 has been observed both in CS [37,38,39,40] and HS [41] GAGs, but in PGs of extracellular matrix tissues, the linker xylose is generally not phosphorylated [42,43,44]. In recent studies, it has been shown that xylose phosphorylation [45] and dephosphorylation [46] are important steps for linker tetrasaccharide completion. The phosphorylation has a maximum at the trisaccharide stage, with two galactoses linked to xylose [47]. The 2-phosphoxylose phosphatase is shown to form hetero-oligomeric complexes with the enzyme adding the fourth linker sugar. This enzyme, the glucuronic acid transferase I (GlcAT-I), aids in Golgi localization and enhances the phosphatase activity [46]. Since xylose phosphorylation is observed for both CS/DS and HS/heparin GAGs, it does not seem to have influence on the type of GAG that will be made, but rather regulates whether a GAG will be polymerized at all, by stimulating enzymes involved in completion of linker tetrasaccharide synthesis [48]. In fact, without xylose phosphorylation, the linker does not seem to proceed with addition of the second galactose unit, but is rather capped by a sialic acid [45].

Sulfation of the linker region is limited to the CS/DS pathway, where 4-*O*-sulfation of the second galactose [49] and 6-*O*-sulfation of both the first and the second galactose units have been shown to occur [50,51,52,53]. Different linker sulfation patterns have been observed for CS/DS from different sources, while as mentioned, linker sulfation has never been observed for HS/heparin chains. A straightforward explanation would be that sulfation of the linker sugars makes a GAG site in a PG protein core bypass the HS synthesis machinery. The enzymes involved in HS synthesis are generally reported to be localized in an earlier region of the Golgi apparatus than the CS polymerizing enzymes. Upon treatment of various cell lines with brefeldin A (BFA) the HS synthesizing enzymes were shown to move retrogradely to the endoplasmic reticulum (ER), while the CS synthesizing enzymes did not [54,55,56,57,58], indicating that the HS enzymes are localized to the BFA-sensitive Golgi *cisternae*, while the CS enzymes are localized to the *trans*-Golgi network (TGN). Mechanistically, the decision in what concerns addition of the fifth sugar unit, which is GalNAc for CS/DS and GlcNAc for HS/heparin, could be more complicated. As mentioned, lack of phosphorylation may lead to capping of the linker region [45], but if the xylose unit is not dephosphorylated before the linker has become a tetrasaccharide, addition of a non-productive GlcNAc unit as the fifth sugar by the enzyme EXTL2 can also prevent GAG elongation [59]. Phosphorylated substrates, with or without 6-*O*-sulfate on the first galactose unit, were better substrates for GlcAT-I [43], but phosphorylated xylosides (xylose linked to a hydrophobic structure) did not promote further linker synthesis [60]. Xylosides, however, might be substrates for other enzymes than those that build on xylose units attached to protein cores [47]. While sulfation of the galactose units of the linker region has been suggested to promote CS/DS synthesis, has 3-*O* sulfation of the fourth sugar unit of the linker (GlcA) been reported to have a rather opposite effect, by preventing CS GAG synthesis on α-thrombomodulin [61]. The enzyme involved may also add 3-*O*-sulfate to the terminal GlcA units of CS chains; therefore it could potentially be a GAG chain termination signal [62]. Variability to the extent of linker region sulfation has been observed since long for tissue preparations [50,63], but the role these modifications might play for the addition of the fifth sugar in the GAG chain has still not been clarified.

## 4. Keratan Sulfate Proteoglycans and Hyaluronic Acid Glycosaminoglycans

Keratan sulfate (KS) GAG chains consist of repeated disaccharides of galactose and GlcNAc, linked together by β(1–4) and β(1–3) linkages, a disaccharide that may also be called polylactosamine. Both units of the disaccharide may be sulfated on the C6 carbon, but GlcNAc sulfation is most abundant. As mentioned, the KSPGs are the only PGs where the GAG chains are not attached to the protein core via the linker tetrasaccharide found in PGs carrying CS, DS, and HS chains, including heparin. The different modes of attachment of KS chains to their protein cores in KSPGs form the basis for the distinction of KSI, KSII and KSIII [10]. For KSI, the GAG chains are linked to the protein core via an asparagine linked (*N*-linked) complex glycan structure, where one (C6), and sometimes both (also C3; [64]), of the *antennae* are further modified by KS. Although it has not been fully determined what protein motifs that instruct a complex *N*-glycan to become KS type I, some aspects have been studied for small leucine-rich corneal PGs. Not all *N*-glycosylation sites may turn into KS [65,66] and those that may seem to have more aromatic residues nearby and localize to an outward facing horseshoe-like structure in the protein core [66]. An *N*-glycosylation site in Aggrecan, however, is reported to carry either KS chains or complex *N*-glycans [67]. KS type II chains are *O*-linked to serine or threonine residues in the protein core via GalNAc units in structures resembling core-2 type mucins. KSII is found in cartilage attached to Aggrecan, predominantly on a serine residue in a repeated Glu-Glu/Lys-Pro-Phe-Pro-Ser sequence, but also elsewhere in the protein core [67,68]. KS type III chains are abundant in the brain and are linked to the protein core via attachment of mannose to serine or threonine residues [69]. The polymerization of KS recruits UDP-galactose from the same pool in the Golgi lumen as does the addition of galactose during formation of complex *N*-linked glycans. This was shown in an MDCK cell line lacking the UDP-galactose transporter in the Golgi membrane [70], which does not synthesize KS or add galactose to *N*-glycans, but synthesizes CS and HS, which requires addition of two galactose units in the linker region, steps thought to occur early in the Golgi apparatus or in a pre-Golgi compartment [71].

Hyaluronic acid (HA), which is a non-sulfated GAG without covalent protein attachment, is abundant in extracellular matrices and at cell surfaces. HA is often bound to PGs called lecticans that possess a HA binding domain called the link protein [72,73]. The monosaccharide building blocks of HA are the same as for HS (GlcA and GlcNAc). In both cases, activation to UDP-sugars in the cytoplasm is required, but while HS chains are polymerized in the Golgi lumen, where they undergo modification by epimerization and sulfation, HA synthases (HAS1-HAS3) are localized to the plasma membrane, recruiting UDP-sugars from the cytoplasmic side, and the precursor pools of HS and HA are therefore segregated [74]. HAS1, HAS2, and HAS3 display different sensitivity to variation in the UDP-sugar levels induced by variable levels of glucose and glucosamine in the cell culture medium [75]. This is to some extent reflected in the estimated K_m_ values for UDP-GlcNAc and UDP-GlcA for these enzymes, which also varies to some extent with the UDP-sugar concentrations [76]. The reported K_m_ values for the UDP-sugars are in the same range as those reported for glycosyltransferases found in the Golgi apparatus (discussed previously; [77]), and 100–1000 times higher than higher than the K_m_ for UDP-GlcNAc of a UDP-GlcNAc transferase acting on cytoplasmic and nuclear proteins [78]. HAS2 is, in fact, a substrate for such modification and *O*-GlcNAcylation stabilizes the enzyme [79], while higher GlcNAc levels also increase the HAS2 V_max_ [76] and the synthesis of both HA and CS [79]. Modification of the enzymes could alter their K_m_ values, which could also be lower for the enzymes in the intact plasma membrane than for individually purified enzymes [80]. Interestingly, HAS1-3 were recently shown to form both homomeric and heteromeric enzyme complexes already in the Golgi apparatus, underway to the cell surface [81].

## 5. Sorting of Proteoglycans in the Secretory Pathway

In eukaryotic cells, the secretory pathway transports secretory and plasma membrane proteins, PGs, and lipids from the ER to the cell surface. In addition, most endosomal and lysosomal proteins follow the secretory pathway from the ER through the Golgi apparatus, from where there are several routes directly to the cell surface, and a number of routes to compartments along the endocytic pathway. In polarized cells, like epithelial cells and neurons, the plasma membrane is divided into different regions, a situation that requires additional pathways for targeting of proteins, PGs and lipids to their respective acceptor membrane domains. The HS chains of Glypican were shown to guide this PG to the basolateral surface of polarized, filter-grown Madin-Darby canine kidney (MDCK) cells, since glypican was transported to a greater extent to the apical cell surface domain upon removal of HS modification sites [82]. It is possible that the HS chains inhibit homoclustering of Glypican molecules in the Golgi apparatus, thereby preventing apical transport [83]. CS chains have been implicated as promoters of apical sorting in MDCK cells. Most of the CSPGs secreted from MDCK cells were recovered from the apical culture medium [84]. An indication that the sorting information could be localized to the CS chains, and not in the protein core, was provided by the finding that protein-free, xyloside-based CS chains were also mainly exported apically [85]. When the PG Serglycin was expressed in MDCK cells, the protein core obtained mainly CS chains and was also secreted predominatly (85%) apically. An interesting finding was that the minor fraction (15%) that was secreted basolaterally carried CS chains that were several times more intensely sulfated than the apical counterpart [86]. In addition, CS chains secreted to the apical and basolateral media were of different lengths [87]. This suggests that Serglycin molecules destined for the apical and basolateral surface domains are segregated during synthesis and modification, making sorting at earlier stages of the secretory pathway than the TGN a possibility [88]. Early segregation would indicate that the required sorting information for apical and basolateral partitioning is present in the protein core or is added in the very first events of modification, for instance in the linker tetrasaccharide sugars and their modifications. When Serglycin was secreted without GAG chains from BFA-treated MDCK cells, presumably from a pre-Golgi compartment, the predominance of apical secretion was maintained, indicative of sorting information localized also elsewhere than, and in addition to, the information in the GAG chains [89].

In fact, a difference in the sulfation intensity in the apical and basolateral pathways of MDCK cells has not only been observed for Serglycin, but also for CSPGs in general [90]. In the case of Serglycin, the GAG attachment domain functioned as an apical sorting signal when it was transferred to the non-glycosylated protein rat growth hormone (rGH; [91]). Interestingly, however, the apically and basolaterally secreted rGH molecules carrying the GAG domains of Serglycin were sulfated with similar intensities. Thus, the higher intensity of basolateral sulfation of the GAGs of intact Serglycin is driven by a region of the protein core outside the GAG-attachment domain [86,91]. In sum, the observations made indicate that information concerning sorting into different secretory routes may be localized to GAG chains and/or their attachment sites, but also to other regions of PG protein cores. Furthermore, a single site for CS modification might not be sufficient to induce apical sorting, since a sole CS GAG chain could not divert the APLP2 splice variant carrying this GAG from the basolateral to the apical secretory route [92]. Single CS chains have, however, been shown to decrease the time of transportation from the TGN to the cell surface, presumably by incorporation into a different transport route [93].

## 6. The Environment of the Golgi Apparatus

Correct organization of the Golgi *cisternae* and their enzymatic content, a shallow pH gradient and proper concentration of ions like Ca^2+^ are requirements for a normal glycosylation output from the secretory pathway. There is also a substrate requirement for the modification reactions that take place within the Golgi *cisternae*, like nucleotide sugars and 3'-phosphoadenosine-5'-phosphosulfate (PAPS), which are transported from their site of synthesis in the cytoplasm into the Golgi lumen through specialized transporters in the Golgi membrane [94,95].

While the ER lumen is neutral, the subsequent intermediate compartment (IC) is slightly acidic (pH 6.7; [96]), followed by a gradually decreasing pH (to 6.3) through the Golgi stacks. The TGN is shown to be more acidic than the Golgi *cisternae* (pH 6.0; [97,98]). A major contributor to the reduced pH within the Golgi apparatus, present from yeast to man [99], is the vacuolar proton translocating ATP-ase (V-ATPase), which is involved in the acidification of endosomes and lysosomes as well [100]. Other Golgi membrane proteins are also required to maintain correct lumenal pH [101,102]. The role of the Golgi lumen pH may be studied by V-ATPase inhibitors or other perturbants. Neutralization of the Golgi lumen changes the glycan output, largely because the proper organization of glycosyltransferases is pH dependent [103,104,105,106]. Increased Golgi pH, accompanied by glycosylation changes, has been reported for diseases like *cutis laxa* [107,108] and for several cancers and cancer cell lines [98,109,110]. Neutralization of the secretory pathway in epithelial MDCK cells resulted in both altered sorting and synthesis of PGs. The dominating basal membrane HSPG was no longer sorted predominantly to the basolateral pole of the cell layer [111], while the differences in apical and basolateral GAG modification observed in untreated MDCK cells were largely abolished upon treatment with the V-ATPase inhibitor Bafilomycin A_1_ [112].

The ER and Golgi apparatus lumens have a high Ca^2+^ level [113], while the TGN consists of two domains with a higher and a lower Ca^2+^ content [114]. The ER Ca^2+^ level has a firmly established function in the quality control system for protein folding, and is also required for the subsequent movement from the ER to the IC and *cis*-Golgi region [115,116,117]. In the TGN, sorting of certain proteins requires a Ca^2+^ binding protein and a Ca^2+^ ATPase [118], and calcium censors regulate TGN to plasma membrane transport of some proteins [119]. Most glycans that modify proteins are negatively charged, particularly the GAG chains of PGs, and Ca^2+^ may serve to coordinate glycan structures during sorting and transport in the secretory pathway [120]. A higher fraction of the cellular HSPGs is present at the cell surface when the extracellular calcium ion level is low [121] and an increase in the extracellular Ca^2+^ also reduced PG secretion in general [122,123]. Depletion of calcium from the lumen of the secretory pathway induced by thapsigargin treatment has been suggested to inhibit the synthesis of both collagen and PGs, while only the secretion of collagen is inhibited [124]. Treatment with the calcium ionophore A23187 also reduced PG synthesis [125]. Calcium ions clearly play a role in cargo sorting in the TGN [126,127], but to what extent calcium ions contribute to PG sorting via association with the GAG chains is unclear at present. The fact that the TGN possesses different domains of high and low calcium ion content points modestly to the fact that the Golgi apparatus is not necessarily a uniform environment at each stage in the *cis* to *trans* direction. In most mammalian cell types, with some exceptions [128], the Golgi stacks are clustered in a ribbon structure in the perinuclear region, where the individual stacks have been difficult to resolve in the confocal microscope. In *Drosophila* imaginal disc cells, individual Golgi stacks are dispersed throughout the cytoplasm. In these cells it was shown that individual stacks displayed different sets of modifying enzymes and the UDP-sugar transporter Fringe-connection, which suggests specialized modification regimes in individual stacks [129]. A similar arrangement may also be possible for mammalian cells, but has not yet been described in detail [88].

## 7. Nucleotide Sugars, PAPS and Their Transporters

The nucleotide sugars and PAPS are synthesized in the cytoplasm, with the exception of CMP-sialic acid, which is produced in the nucleus. The synthesis requires energy; for instance does the production of one PAPS molecule require one sulfate ion and two molecules of ATP [130]. As the nucleotide sugars and PAPS are needed in glycosylation and sulfation mechanisms that take place in the Golgi lumen, they must be transported through specific carriers in the Golgi membrane, in exchange for nucleoside monophosphates (like UMP) by an antiport mechanism [131,132]. These transporters in the Golgi membrane have been studied to a lesser extent than the modifying enzymes in the Golgi lumen, but the supply of both UDP-sugars [133] and PAPS [134,135] has been shown to be essential for development. The import of substrate seems to be a rate limiting step, since additional expression of PAPS transporters [90] enhances PAPS uptake and the subsequent utilization, while an increase in the availability of UDP-N-acetylhexosamine (UDP-GlcNAc and -GalNAc), lead to enhancement of the incorporation into glycoconjugates [136]. This suggests that several Golgi enzymes normally operate at sub-optimal substrate concentrations. Determination of such substrate concentrations in the intact Golgi is difficult, however, since such biochemical assays are conducted with isolated vesicle fractions, and the concentration of the substrates might not be uniform across the whole Golgi apparatus. The dilatation observed in the presence of monensin (10 µM) gives the Golgi compartment a larger volume and leads to enhanced accumulation of nucleotide-sugars [137].

The substrate availability in the Golgi lumen must to some extent reflect the situation in the cytoplasm. Further, a single UDP-sugar transporter has been reported for UDP-glucose, UDP-galactose, UDP-GlcNAc, and UDP-GalNAc in the nematode *Caenorhabditis elegans* [138]. Some redundancy of UDP-sugar transport has also been observed in mammalian cells, indicating that some competition for uptake into the Golgi lumen among different UDP-sugars is possible [139]. Furthermore, while epimerases that operate at the level of nucleotide sugars have been identified in the Golgi lumen of plant cells [140], such enzymatic activities have not been reported in the Golgi apparatus of mammalian cells. The only enzyme so far found in the mammalian Golgi that converts UDP-sugars is a UDP-GlcA decarboxylase, which produces UDP-xylose [141].

The possibility that the Golgi apparatus is organized in subdomains containing modifying enzymes [142], or in segregated transport and modification routes [88,129], may require some co-ordination of modifying enzymes and nucleotide sugar transporters [143,144]. In fact, it has recently been shown that the transporters for UDP-galactose and UDP-GlcNAc form heterologous complexes in the proximity of GlcNAc-transferases [145].

## 8. Conclusions and Future Perspectives

It is well established that motifs in the PG protein cores influence the structure of the GAG chains that are attached to their potential modification sites. In light of the current knowledge the protein cores seem to be sorted into a suitable Golgi environment where the correct modifying enzymes are encountered and assembled. An issue that has not been much studied is whether or not the sites of GAG modification are 100% occupied. In the case of *N*-glycosylation, the site occupancy is variable and rarely 100% [146]. Some indications exist that PG protein cores sometimes operate without their GAG chains [147,148]. Although PG protein cores may be decisive for their own GAG structures, individual GAGs extending from xylosides may be variable in type and structure [149]. Xylosides consist of xylose units that are linked to sufficiently hydrophobic moieties that will allow for transport across the plasma and Golgi membranes. Different xylosides may possibly localize to different Golgi membrane regions or their GAG linker regions may recruit different modifying enzymes. The organization of Golgi enzymes involved in GAG synthesis is still under investigation [150]. While several Golgi enzymes involved in glycoprotein synthesis form homomeric complexes in the ER, but convert to heteromeric complexes in the Golgi apparatus [151], EXT1 and EXT2, the enzymes involved in HS polymerization, must associate in the ER to move forward to the Golgi apparatus [152]. In addition, EXT1 and EXT2 influence the expression of *N*-deacetylase/*N*-sulfotransferase (NDST) in opposite ways, indicating complex modes of association [153]. In S2 cells from *Drosophila*, the glucuronyl C5-epimerase, converting GlcA to IdoA in HS/heparin chains, is in a complex with enzymes mediating 2-*O*-sulfation and 6-*O*-sulfation of HS chains [154]. Thus, there is some evidence favoring the existence of GAGosome complexes of enzymes involved in PG synthesis [149,155], but co-localization of the enzymes in the Golgi apparatus needs to be demonstrated. The organization of the enzymes and transporters required for GAG synthesis in the Golgi apparatus should be a topic for intensified research activity in the future.
